# Recalling visual serial order for verbal sequences

**DOI:** 10.3758/s13421-015-0580-9

**Published:** 2015-12-24

**Authors:** Robert H. Logie, Satoru Saito, Aiko Morita, Samarth Varma, Dennis Norris

**Affiliations:** Human Cognitive Neuroscience, Centre for Cognitive Ageing and Cognitive Epidemiology, University of Edinburgh, 7 George Square, Edinburgh, EH16 6JF UK; Department of Cognitive Psychology in Education, Kyoto University, Kyoto, Japan; Department of Psychology, Hiroshima University, Hiroshima, Japan; Human Cognitive Neuroscience, Department of Psychology, University of Edinburgh, Edinburgh, UK; MRC Cognition and Brain Sciences Unit, Cambridge, UK

**Keywords:** Short-term memory, Working memory, Serial position effects, Visual similarity

## Abstract

We report three experiments in which participants performed written serial recall of visually presented verbal sequences with items varying in visual similarity. In Experiments 1 and 2 native speakers of Japanese recalled visually presented Japanese Kanji characters. In Experiment 3, native speakers of English recalled visually presented words. In all experiments, items varied in visual similarity and were controlled for phonological similarity. For Kanji and for English, performance on lists comprising visually similar items was overall poorer than for lists of visually distinct items across all serial positions. For mixed lists in which visually similar and visually distinct items alternated through the list, a clear “zig-zag” pattern appeared with better recall of the visually distinct items than for visually similar items. This is the first time that this zig-zag pattern has been shown for manipulations of visual similarity in serial-ordered recall. These data provide new evidence that retaining a sequence of visual codes relies on similar principles to those that govern the retention of a sequence of phonological codes. We further illustrate this by demonstrating that the data patterns can be readily simulated by at least one computational model of serial-ordered recall, the Primacy model (Page and Norris, Psychological Review, 105(4), 761–81, 1998). Together with previous evidence from neuropsychological studies and experimental studies with healthy adults, these results are interpreted as consistent with two domain-specific, limited-capacity, temporary memory systems for phonological material and for visual material, respectively, each of which uses similar processes that have evolved to be optimal for retention of serial order.

## Introduction

Temporary retention of serial order (e.g., of actions, objects, or words) is fundamental to a wide range of cognitive tasks. The majority of studies of immediate serial-ordered recall have used verbal stimuli where the assumption is that the underlying codes for the sequences are phonological or speech-based, regardless of whether presentation is visual or auditory. However, in recent years there has been debate as to the nature of the cognitive mechanisms that might support retention of serial order. In their review of research on verbal serial order, Hurlstone, Hitch and Baddeley (2014) note that there is a lack of studies that have explored the use of visual codes in serial recall tasks. In the three experiments reported here, we explored whether serial recall of visually presented verbal lists might involve the use of visual as well as verbal codes, whether the characteristics of serial recall are similar regardless of the type of code used, and whether those characteristics are the same in a logographic (Japanese Kanji) and an alphabetic (English) language.

In our previous work, we have demonstrated poorer written serial recall of lists of visually similar compared with visually distinct English words and letters when phonological similarity is controlled (Logie, Della Sala, Wynn & Baddeley, 2000). In a later study Saito, Logie, Morita and Law (2008) demonstrated independent and additive effects of visual similarity and phonological similarity within the same stimulus lists when both forms of similarity were manipulated orthogonally for Japanese kanji characters. Moreover, the phonological similarity effect was removed by concurrent articulation, but the visual similarity effect remained intact, or was enhanced when articulatory rehearsal was prevented. Similar findings of independent phonological and visual similarity effects, and selective disruption of phonological but not visual similarity by concurrent articulation, have been reported recently by Lin, Chen, Lai and Wu (2015) using a probe technique to test memory for serial order of Chinese characters. Further evidence for the use of visual codes in serial recall tasks comes from the finding that serial recall of sequences of matrix patterns (Avons & Mason, 1999; Walker, Hitch & Duroe, 1993), and of faces (Smyth, Hay, Hitch & Horton, 2005) is disrupted when stimuli are visually similar. Guérard, Neath, Surprenant and Tremblay (2010) reported a visual distinctiveness effect in recall of non-verbal spatial sequences. Poirier, Saint-Aubin, Musselwhite, Mohanadas and Mahammed (2007) reported evidence for the use of both phonological and visual codes in memory for serial order of line drawings that were easily nameable and for more abstract matrix patterns. Collectively, this previous evidence suggests that both phonological and visual codes can be used to support immediate serial recall of visually presented verbal as well as non-verbal material.

The above findings, and particularly the findings from Saito et al. (2008) and Lin et al. (2015), are consistent with neuropsychological evidence that immediate serial recall based on phonological codes and immediate serial recall based on visual codes might rely on separate, domain-specific temporary memory stores. For example, there are several reports of brain-damaged individuals with a specific impairment of verbal serial-ordered recall who also fail to show disruptive effects of phonological similarity with auditory presentation. Typically, such patients can recall more items in the correct serial order when items are presented visually than when they are presented aurally. With visual presentation, errors are based on visual similarity of the items, and levels of performance are similar to those for healthy adults performing the same task with concurrent articulation (e.g., Basso, Spinnler, Vallar & Zanobio, 1982; Beyn & Knyazeva, 1962; Shallice & Warrington, 1970; Warrington & Rabin, 1971; Warrington & Shallice, 1972; for reviews see Vallar & Shallice, 1990; Logie, 1995; Logie & Della Sala, 2005). This suggests that an intact temporary visual store might be able to support serial recall performance, even if there is damage to the system that supports serial order for phonological codes, or the use of the latter is prevented by concurrent articulation in healthy adults. It is also possible that healthy adults might simply choose to retain items using visual rather than phonological codes in immediate serial-ordered recall tasks with visual presentation (e.g., Logie, Della Sala, Laiacona, Chalmers & Wynn, 1996; Della Sala, Logie, Marchetti & Wynn, 1991). Together, the previous evidence from healthy adults and from neuropsychological studies demonstrates that visual temporary serial-ordered memory and phonological temporary serial order memory can be damaged independently, can be disrupted independently in healthy adults, and can contribute additively to serial-ordered recall performance.

What remains unclear is whether temporary memory for ordered sequences of visual codes involves similar processes to retention of ordered sequences of phonological codes. This issue is particularly important given the evidence described above that the use of different codes might involve separate, domain-specific temporary memory systems. There is some evidence for such similar processes. For example, Avons (1998; Avons & Mason, 1999) reported that when memory for visual serial order was tested by having participants select items in order from a test array of visual patterns, recall showed a bowed serial position curve with both primacy and recency effects. The bowed serial position function has also been shown using this same serial reconstruction technique with sequences of faces (Smyth et al., 2005). Avons (1998), Avons and Mason, (1999), and Smyth et al. (2005) raised the possibility that the same system might support retention of serial order regardless of whether the material is visual or phonological. An alternative view, also raised by Smyth et al. (2005) and Saito et al. (2008), is that any system supporting memory for serial order might show characteristic serial position curves and effects of within-list item similarity, even if there are separate, modality-specific temporary memory systems. It may indeed be the case that there is an optimal algorithm for retention of serial order in any temporary memory system, although a detailed discussion of this issue is outside the scope of the current paper.

Here we explore further whether retention of visual serial order shows the same performance characteristics as have been found in previous studies of phonological serial order by examining memory for visual sequences that vary in their pattern of visual similarity. For this, we have drawn on seminal research on phonological similarity in serial recall. Baddeley (1968) studied phonological similarity using lists of letters comprising alternating phonologically similar and dissimilar items within each presented list for recall, as well as lists consisting entirely of phonologically similar or phonologically dissimilar items. There was poorer performance on the phonologically similar items relative to the phonologically dissimilar items, even in the alternating lists where similar items were not adjacent in the list. This last finding was evident in a characteristic “saw-tooth” pattern when plotting the recall data for alternating similar and dissimilar items and across serial positions. Baddeley also found that performance on dissimilar items in alternating lists was no different from that in pure lists of dissimilar items. This result is important because it appears to rule out models of memory for serial order that rely on a simple chain of associations between successive items (for a review and discussion see Hurlstone et al., 2014). According to a simple chaining model, if there is an error on one item, that item should not provide a reliable cue for the retrieval of the next item, and the error should propagate throughout the list. However, Baddeley’s data showed that memory for dissimilar items in alternating lists is unaffected by whether the immediately preceding items is an accurately recalled dissimilar item or a less well recalled similar item. These results were replicated and extended by Henson, Norris, Page and Baddeley (1996).

Our goal here is to address the lacuna in research on visual serial recall noted by Hurlstone et al. (2014) by following up on the Logie et al. (2000) and Saito et al. (2008) studies on this topic. We explored across three experiments whether the characteristic saw-tooth data pattern for alternating lists might also be present in recall of items that alternate visually similar and visually distinct verbal items that are presented visually. Note that neither in our previous work (Logie et al., 2000; Saito et al., 2008) nor here do we suggest that the use of visual codes is obligatory for healthy adults (e.g., Fürstenberg, Rummer & Schweppe, 2013). Rather we are suggesting that visual codes are available and may be used strategically along with other information about the stimulus set to support retention and retrieval of serial order (Logie et al., 1996).

We investigated the retention of serial order by means of visual codes for visually presented verbal material by manipulating whether list types (mixed visually similar and visually different items or pure lists of one item type) were randomized within blocks of trials (Experiment 1), broadly following the design in Baddeley (1968), or each trial block comprised the same list type (Experiments 2 and 3), following the general design used by Henson et al. (1996). We anticipated that visual similarity effects would be more evident with blocked lists. In blocked lists participants might be more likely to establish an encoding strategy that they would apply consistently across lists. In contrast, in mixed blocks when participants cannot anticipate which list type is going to be presented next, they would be more likely to swap between code types, or default to attempting the use of phonological codes.

Finally, the Logie et al. (2000) experiments used English language materials, whereas the Saito et al. (2008) experiments used Japanese Kanji materials. In the experiments reported here, we used equivalent paradigms in the two languages with native speakers of each: Experiments 1 and 2 with Japanese Kanji, and Experiment 3 with the English language. In part this was because Kanji involves ideograms rather than a phonetic alphabet and may therefore be more likely to encourage the use of visual codes, even in native speakers of Japanese. We wished to explore whether our findings are universal for serial-ordered recall of visual codes across very different written languages rather than specific to Japanese. In each case participants performed serial-ordered written recall of visually presented sequences of verbal stimuli that varied in visual similarity but were controlled for phonological similarity. Item frequency was controlled, and the influence of semantic content was minimized by using semantically unrelated words. The visual writing complexity of Kanji words was also controlled. Concurrent articulation was used to discourage the use of phonological coding. In each experiment we used a small pool of items, with any one participant seeing the same items repeated in different orders across trials. This was to reduce the possibility of extra-list intrusions in recall and to help reduce the contribution of item-specific information to the retention of serial order, thereby allowing a focus on the manipulations of phonological and visual similarity for immediate serial-ordered recall. We recognize that use of small word pools may reduce but might not completely eliminate item-specific contributions (e.g., Lin et al., 2015; Taylor, Macken & Jones, 2015). However, different small pools of items were used for each experiment, two in Kanji (modified from Saito et al., 2008) and one in English (based on Logie et al., 2000), to ensure that any findings cannot be attributed to the idiosyncracies of a specific language or a specific item set.

To summarize, previous experimental and neuropsychological evidence points to the possibility that there are modality-specific temporary memory systems for retention of, respectively, phonological codes and visual codes in serial order. The systems can be damaged independently, and disrupted independently in healthy adults. What remains unclear and has not been investigated in previous studies is whether temporary memory for ordered sequences of visual codes involves similar processes to retention of ordered sequences of phonological codes. We tested this by exploring whether serial-ordered recall of lists with alternating visually similar and visually different items results in the characteristic zig-zag pattern that has previously been reported for alternating lists of phonologically similar and phonologically different items.

## Experiment 1

### Method

#### Participants

Thirty undergraduate and postgraduate students from Hiroshima University participated in this experiment. They were all native speakers of Japanese, and their age ranged from 19 to 28 years, with a mean of 20.2 years.

#### Design and materials

Each experimental block comprised six list conditions as follows: Pure dissimilar lists in which all six items were visually dissimilar from each other (DDDDDD), pure similar lists in which all six items were visually similar to each other (SSSSSS), DS alternating lists in which visually dissimilar and visually similar items alternated starting from a dissimilar item (DSDSDS), SD alternating lists which started from a similar item (SDSDSD), DS combined lists in which the first three items were visually dissimilar but the second half were visually similar items (DDDSSS), and SD combined lists in which the first three items were similar and the second half were dissimilar (SSSDDD).

All materials were single Japanese words represented by a single Kanji character. They had a bimoraic structure in spoken format (CV-CV or V-CV). The mora is a subsyllabic unit in Japanese. It can be a vocalic nucleus (V), a nucleus with onset (CV or CCV), or a nasal consonant (N) in syllabic coda position (Cutler & Otake, 1994). Japanese is considered a mora-rhythm language rather than a language based on a syllabic or stress-based rhythm (e.g., McQueen, Otake & Cutler, 2001; Otake, Hatano, Cutler, & Mehler, 1993).

Six single Kanji words were selected for each of the two sets (visually dissimilar and visually similar), thus 12 Kanji characters in total. In order to control phonological similarity within each set, both visually dissimilar and visually similar items were phonologically dissimilar. Because it was difficult to create a stimulus set in which all six characters were visually similar, we divided the stimulus set of six words into two subsets of three words and created the subsets in which all combinations among three words followed the definition of this condition (i.e., phonologically dissimilar but visually similar). In the visually dissimilar set, a stimulus set of six words also consisted of two subsets of three words, which were arranged in a similar manner to the similar set. The definition of visual similarity was whether or not a pair of two characters shared a radical. We ensured that items in each subset were phonologically dissimilar by not repeating the same mora within each subset. Also, we attempted to select words that were not semantically related to each other. Most of the items were selected from the phonologically dissimilar materials used in Experiment 3 of Saito et al. (2008).

We also attempted to control frequency of the Kanji words and the number of strokes for each character, with the latter used as an index of visual and/or writing complexity. Mean log-transformed frequency of the Kanji words in each condition is shown in Table 1. The mean number of strokes and the mean age of acquisition (AoA) of the Kanji words in each condition are also shown in Table 1. One-way ANOVAs showed no significant differences between similar and dissimilar sets for the frequency, the number of strokes, or AoA. Kanji letters, meanings, pronunciations, the numbers of strokes, log frequency, and age of acquisition for all stimuli are listed in Table 1.

**Table 1:** Kanji materials used in Experiment 1, listed with visual form, meaning, pronunciation, number of strokes, log-transformed frequency, and age of acquisition (AoA). Kanji frequency counts are based on a 1-year volume of the Asahi Newspaper (Yokoyama et al., 1998; The National Language Research Institute in Japan). AoA values are ages at which the Kanji words are officially taught in school, based on the national curriculum in Japan

	Kanji	Meaning	Pronunciation	Strokes	Log-transformed frequency	AoA
Visually dissimilar	姓	Family name	*se-i*	8	2.748	12
	草	Grass	*ku-sa*	9	3.485	6
	並	Common quality	*na-mi*	8	3.844	11
	夜*	Night	*yo-ru*	8	3.826	7
	丘	Hill	*o-ka*	5	2.992	12
	枠	Frame	*wa-ku*	8	3.467	12
Mean				7.67 (7.33)	3.393 (3.345)	10.00 (10.83)
Visually similar	仮	Temporary	*ka-ri*	6	3.307	10
	坂	Slope	*sa-ka*	7	3.595	8
	板	Board	*i-ta*	8	3.483	8
	汁	Soup	*shi-ru*	5	2.800	12
	肝	Liver	*ki-mo*	7	3.176	12
	汗	Sweat	*a-se*	6	2.670	12
Mean				6.50	3.172	10.33

*Note: This word is replaced by another word 旬 (shu-n), meaning “in season” for better control of the number of strokes (6), frequency (3.535), and age of acquisition (12) in Experiment 2. Resultant mean values for the visually dissimilar set are shown in parentheses in this table

#### Procedure

Participants in this and all subsequent experiments were tested individually. Before performing the memory task they engaged in a Kanji reading task. This task was required in order to ensure that each participant could correctly pronounce each Kanji word. The 12 Kanji words were presented on a computer display one at a time, and participants had to read aloud each presented word. In the few cases where a participant produced a pronunciation that was not expected (most Kanji characters have multiple pronunciations with each depending on context), the pronunciations were corrected by an experimenter.

##### Memory task

On each trial participants were shown a sequence of six Kanji words, one at a time, at the center of a computer display. The six words were from two subsets of three words. For pure lists, the two subsets were from the same set (visually dissimilar or visually similar). For alternating lists and combined lists the two subsets were from different sets: One from the dissimilar, another from the similar set. The task of the participants was to read each word silently and to remember the order of the six words. When participants were ready to begin, the experimenter pressed a key, which terminated the presentation of a fixation point and displayed the first word on a display controlled by a personal computer (OteckIdaten Neo-I 7500X). The words were presented sequentially for 1 s each on the display. The list was followed by a question mark which signalled the start of the recall period. Written strict serial recall was used. The participants wrote their responses from left to right on a recall sheet that contained six blank boxes. They were not allowed to retrace leftward to change their answer. A new recall sheet was provided for each trial.

##### Concurrent articulation

In all conditions participants performed the serial recall task under concurrent articulation in order to minimize the use of phonological codes. The concurrent articulation procedure involved participants uttering aloud “1, 2, 3, 1, 2, 3…” from the onset of the fixation point, which signalled the start of the trial, until the end of recall period. Thus, suppression was required during both presentation and recall, following Saito et al. (2008). The participants were encouraged to repeat “1, 2, 3” at a rate of approximately three digits per second and were cautioned if their rate of articulation showed signs of slowing. During the practice phase, the experimenter closely monitored participants’ behavior and, if necessary, reminded them to perform the concurrent articulation task at an appropriate pace.

Thirty-six lists (six for each of the six list types) were presented in random order for each participant. Before they began the 36 experimental trials, participants were given six practice trials, one for each type, presented in a random order, following the presentation procedure used by Baddeley (1968).

### Results

#### Scoring methods

Recall of the Kanji characters was scored in three ways following the analysis procedures used by Walker and Hulme (1999) who examined item information and order information separately in serial order recall. First, correct recall score was based on correct recall of items in the serial position in which they were presented. The total number of correct-in-position recall items was converted into proportion of correct recall at each serial position. It is possible that the correct recall scores might be influenced by both item (omission and intrusion) errors and by order errors in recall. To explore whether our pattern of findings arose from item-based information or from retention of serial order, the second scoring method focused on mean proportions of item errors, that were given as the proportions of the total number of items presented in each condition (and at each presented position). Finally, given that our focus was on the role of visual codes in retention of serial order, our third and key measure of performance indicated memory for order. The proportion of order errors was calculated by dividing the number of items recalled in the wrong serial position by the total number of items recalled (Walker & Hulme, 1999).

In statistical analyses of each scoring method we collapsed over serial position and compared scores from visually dissimilar and similar items within each of the list structures after calculating the scores in each serial position. Thus, we collapsed scores across the two alternating lists (SDSDSD and DSDSDS) and collapsed scores across the two combined lists (SSSDDD and DDDSSS), and then compared performance on the similar and dissimilar items for each list combination. Consequently, the following analyses are based on a design that included two within-participant factors: Visual similarity (dissimilar vs. similar) and list structure (pure, alternating, and combined lists). However, for the sake of complete reporting, mean data are presented in tables and figures separately for each individual list type.

#### Correct recall

Table 2 shows the mean proportion of correct recall as a function of serial position in each of the six list types. We conducted a two-way analysis of variance (ANOVA), with visual similarity factor (two levels: dissimilar vs. similar) and list structure factor (three levels: pure, alternating, and combined lists). Mean scores were .53, .51, .49, 43, 58, and .56, respectively for Dissimilar-Pure, Similar-Pure, Dissimilar-Alternating, Similar-Alternating, Dissimilar-Combined, and Similar-Combined lists. The results of the ANOVAs are reported in Table 3 on all three scores for each experiment. The main effect of similarity was marginal, indicating possible better recall performance for dissimilar over similar items. The main effect of list structure was significant but the interaction between the two factors was not significant.

**Table 2:** Proportions of correct recall in Experiments 1, 2, and 3

List type		Serial position						
		1	2	3	4	5	6	Total
Experiment 1: Random list presentation in Japanese								
DDDDDD	M	0.844	0.711	0.483	0.433	0.339	0.378	0.531
	SD	0.210	0.255	0.291	0.272	0.249	0.239	0.189
SSSSSS	M	0.806	0.622	0.517	0.344	0.328	0.422	0.506
	SD	0.240	0.251	0.237	0.223	0.249	0.279	0.174
DSDSDS	M	0.806	0.567	0.517	0.289	0.306	0.294	0.463
	SD	0.191	0.268	0.249	0.243	0.240	0.258	0.168
SDSDSD	M	0.711	0.578	0.417	0.350	0.322	0.356	0.456
	SD	0.200	0.209	0.195	0.278	0.277	0.218	0.147
DDDSSS	M	0.833	0.717	0.611	0.489	0.394	0.378	0.570
	SD	0.186	0.248	0.267	0.227	0.212	0.210	0.164
SSSDDD	M	0.783	0.644	0.678	0.483	0.394	0.439	0.570
	SD	0.232	0.254	0.231	0.278	0.275	0.242	0.172
Experiment 2: Blocked list presentation in Japanse								
DDDDDD	M	0.846	0.738	0.667	0.533	0.483	0.525	0.632
	SD	0.132	0.147	0.206	0.186	0.241	0.254	0.139
SSSSSS	M	0.767	0.654	0.550	0.454	0.458	0.508	0.565
	SD	0.204	0.252	0.228	0.172	0.222	0.224	0.171
DSDSDS	M	0.779	0.579	0.579	0.446	0.496	0.392	0.545
	SD	0.179	0.228	0.252	0.177	0.274	0.189	0.177
SDSDSD	M	0.804	0.742	0.575	0.513	0.508	0.513	0.609
	SD	0.176	0.224	0.217	0.221	0.243	0.175	0.164
Experiment 3: Blocked list presentation in English								
DDDDDD	M	0.694	0.535	0.507	0.417	0.465	0.549	0.528
	SD	0.223	0.214	0.205	0.246	0.251	0.211	0.101
SSSSSS	M	0.653	0.465	0.389	0.382	0.410	0.507	0.468
	SD	0.196	0.203	0.153	0.271	0.230	0.243	0.112
DSDSDS	M	0.736	0.451	0.472	0.333	0.493	0.417	0.484
	SD	0.303	0.297	0.259	0.269	0.238	0.184	0.115
SDSDSD	M	0.604	0.618	0.340	0.431	0.389	0.604	0.498
	SD	0.250	0.228	0.211	0.240	0.317	0.219	0.131

**Table 3:** Outcomes of the ANOVAs for each scoring method in each experiment

		Correct recall				Item errors				Order errors			
		*df*	*F*	*η* ^*2*^_*G*_	*p*	*df*	*F*	*η* ^*2*^_*G*_	*p*	*df*	*F*	*η* ^*2*^_*G*_	*p*
Exp. 1	List type	2	16.735	0.069	.000 **	2	61.736	0.154	.000 **	2	2.600	0.016	.083 +
	Error	58				58				58			
	Similarity	1	4.011	0.009	.055 +	1	6.288	0.033	.018 *	1	16.557	0.057	.005 **
	Error	29				29				29			
	Interaction	2	0.556	0.002	.576	2	2.099	0.009	.132	2	0.557	0.003	.576
	Error	58				58				58			
Exp. 2	List type	1	0.645	0.004	.430	1	10.496	0.024	.004 **	1	0.146	0.001	.706
	Error	23				23				23			
	Similarity	1	7.938	0.033	.009 **	1	0.011	0.000	.916	1	9.508	0.044	0.005 **
	Error	23				23				23			
	Interaction	1	0.095	0.000	.760	1	0.209	0.001	.652	1	0.080	0.000	.780
	Error	23				23				23			
Exp. 3	List type	1	0.094	0.001	.762	1	22.906	0.193	.000 **	1	4.008	0.043	.057 +
	Error	23				23				23			
	Similarity	1	43.346	0.167	.000 **	1	10.437	0.065	.004 **	1	17.560	0.115	.000 **
	Error	23				23				23			
	Interaction	1	11.277	0.032	.003 **	1	21.081	0.123	.000 **	1	0.037	0.000	.849
	Error	23				23				23			

** p < .01, * p < .05, + p < .10

Note: η^2^_G_ = generalized η^2^ (Olejnik & Algina, 2003)

#### Item errors

Table 4 shows the mean proportion of item errors as a function of serial position in each list of six list types. A two-way ANOVA, with factors of visual similarity (two levels: dissimilar vs. similar) and list structure (three levels: pure, alternating, and combined lists) showed a significant main effect of similarity but with similar items being better recalled than dissimilar items. The main effect of list structure was significant, but the interaction between the two factors was not. The mean proportions of item errors for Dissimilar-Pure, Similar-Pure, Dissimilar-Alternating, Similar-Alternating, Dissimilar-Combined, and Similar-Combined lists were .23, .19, .32, 30, 22, and .13, respectively.

**Table 4:** Proportions of item errors in Experiments 1, 2, and 3

List type		Serial position							
		1	2	3	4	5	6		Total
Experiment 1: Random list presentation in Japanese									
DDDDDD	M	0.056	0.094	0.256	0.272	0.372	0.344		0.232
	SD	0.119	0.129	0.239	0.264	0.283	0.195		0.130
SSSSSS	M	0.072	0.122	0.178	0.228	0.311	0.222		0.189
	SD	0.136	0.175	0.200	0.238	0.283	0.202		0.169
DSDSDS	M	0.100	0.217	0.294	0.350	0.417	0.433		0.302
	SD	0.143	0.215	0.213	0.278	0.222	0.242		0.122
SDSDSD	M	0.194	0.272	0.283	0.417	0.322	0.433		0.320
	SD	0.158	0.238	0.219	0.290	0.227	0.189		0.119
DDDSSS	M	0.067	0.133	0.211	0.161	0.211	0.161		0.157
	SD	0.104	0.188	0.231	0.188	0.219	0.208		0.134
SSSDDD	M	0.044	0.133	0.078	0.256	0.311	0.317		0.190
	SD	0.097	0.154	0.114	0.204	0.235	0.207		0.099
Experiment 2: Blocked list presentation in Japanese									
DDDDDD	M	0.038	0.063	0.025	0.021	0.063	0.063		0.045
	SD	0.065	0.082	0.061	0.051	0.106	0.110		0.055
SSSSSS	M	0.042	0.067	0.042	0.054	0.025	0.075		0.051
	SD	0.088	0.137	0.110	0.106	0.053	0.115		0.087
DSDSDS	M	0.038	0.083	0.075	0.050	0.096	0.104		0.074
	SD	0.058	0.120	0.090	0.083	0.112	0.127		0.068
SDSDSD	M	0.038	0.046	0.071	0.113	0.071	0.071		0.068
	SD	0.092	0.072	0.130	0.130	0.130	0.100		0.085
Experiment 3: Blocked list presentation in English									
DDDDDD	M	0.111	0.201	0.264	0.271	0.313	0.236		0.233
	SD	0.117	0.184	0.214	0.195	0.247	0.230		0.101
SSSSSS	M	0.111	0.229	0.250	0.243	0.250	0.174		0.209
	SD	0.136	0.183	0.184	0.255	0.231	0.217		0.120
DSDSDS	M	0.167	0.285	0.271	0.500	0.333	0.465		0.337
	SD	0.214	0.211	0.195	0.278	0.220	0.184		0.078
SDSDSD	M	0.250	0.160	0.451	0.313	0.354	0.285		0.302
	SD	0.214	0.143	0.211	0.198	0.252	0.193		0.069

#### Order errors

**Fig. 1:**
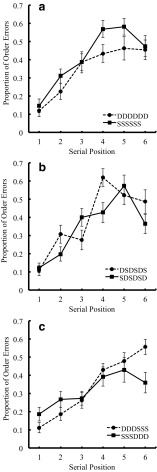
Serial position curves in the proportion of order errors in Experiment 1, as a function of visual similarity for pure lists (Fig. 1a), for alternating lists (Fig. 1b), and for combined lists (Fig. 1c). The data are from 30 participants. However, due to the presence of zero correct item recall at some presentation positions (where the denominator becomes zero for proportion of order errors), the number of participants at serial position 4 of pure dissimilar lists and position 5 of pure similar lists were 29 in Fig. 1a, and that at serial position 4 of SD-alternating lists was 28 in Fig. 1b

We examined the order error data for each condition. Figure 1a shows serial position curves for the proportion of order errors for the pure lists (pure lists for visually dissimilar vs. similar), Fig. 1b shows those of the alternating lists (DS alternating and SD alternating lists), and Fig. 1c shows those of the combined lists (DS combined and SD combined lists). A two-way ANOVA, with visual similarity factor (two levels: dissimilar vs. similar) and list structure factor (three levels: pure, alternating, and combined lists) revealed a significant main effect of visual similarity, indicating superior recall performance for dissimilar over similar items. A main effect of list structure was marginal. The interaction between the two factors was not significant. The mean proportion of order errors for Dissimilar-Pure, Similar-Pure, Dissimilar-Alternating, Similar-Alternating, Dissimilar-Combined, and Similar-Combined lists were .35, .41, .31, .42, .29, and .37, respectively.

In order to test for the presence of saw-tooth serial position curves, we conducted a two-way ANOVA, with list type (DS alternating vs. SD alternating lists) and serial position (1–6), resulting in a significant interaction between the two factors. The results of the ANOVA and the results of analyses of simple main effects for each serial position are reported in Table 5 along with the results of similar analyses from the other two experiments. Simple main effects show that the visual similarity effect was not significant at all serial positions, although near ceiling performance at position 1, and close to floor performance at position 5 could have masked any effect at those positions. Nevertheless, the overall significant interaction confirms the sawtooth serial position curves.

**Table 5:** Outcomes of the two-way ANOVAs for the alternating lists (DSDSDS vs. SDSDSD) on order errors in each experiment

		ANOVAs				Simple main effects: List type by serial position		
		*df*	*F*	*η2G*	*p*	Serial position	*p*	Direction of the effect on errors
Exp. 1	List type	1	0.234	0.001	.632	1	.891	-
Fig. 1b	Error	27				2	.286	-
	Serial position	5	15.253	0.154	.000 **	3	.046*	Similar > Dissimilar
	Error	135				4	.012*	Similar > Dissimilar
	Interaction	5	4.540	0.038	.012 *	5	.139	-
	Error	135				6	.294	-
Exp. 2	List type	1	7.748	0.025	.011*	1	.557	-
Fig. 2b	Error	23				2	.002**	Similar > Dissimilar
	Serial position	5	33.437	0.235	.000 **	3	.737	-
	Error	115				4	.018*	Similar > Dissimilar
	Interaction	5	0.095	0.022	.027*	5	.889	-
	Error	115				6	.006**	Similar > Dissimilar
Exp. 3	List type	1	0.011	0.000	.918	1	.562	-
Fig. 3b	Error	23				2	.049*	Similar > Dissimilar
	Serial position	5	4.689	0.080	.001 **	3	.925	-
	Error	115				4	.426	-
	Interaction	5	3.947	0.042	.002 **	5	.002**	Similar > Dissimilar
	Error	115				6	.165	-

** p < .01, * p < .05, + p < .10

Note: η2G = generalized η2 (Olejnik & Algina, 2003)

### Discussion

This experiment demonstrated zig-zag serial position curves for the proportion of order errors. In the alternating lists the advantage for visually dissimilar items was maintained despite them being adjacent to visually similar items. Moreover, this visual similarity effect appeared as a main effect, regardless of list type. This pattern of results for visual similarity mirrors that observed by Baddeley (1968) and Henson et al. (1996) for phonological similarity, and suggests that similar mechanisms may be involved in the retention of visual and phonological serial order. The result also matches that found for pure lists in our previous study (Saito et al., 2008). It is notable that although statistically reliable in this first experiment for order errors, the size of the visual similarity effect (generalized η^2^ or η^2^_G_ = .057 in this experiment; η^2^_G_ = .039 in Saito et al., 2008) is in general smaller than that of the phonological similarity effect found in our own (η^2^_G_ = .099 in Saito et al., 2008) and in other studies, and the effect was marginal for the correct recall score. Although we found that order recall was impaired by visual similarity, item recall was enhanced by similarity. This pattern of results has been reported in studies of phonological similarity (e.g., Fallon, Groves & Tehan, 1999; Gupta, Lapinski & Actunc, 2005) and has generally been attributed to the potential for shared properties of the stimuli to provide additional cues for item recall. For example, in the present experiment the presence of shared radicals in similar lists might provide an additional recall cue for item recall, but this would be of little value in enhancing recall of order.

Because the effect of visual similarity is small it is likely to be susceptible to influences of strategic changes (e.g., Logie et al., 1996) that might eliminate the effect. As mentioned in the Introduction, the structure of the randomized-list blocks might possibly lead participants to use variable strategies across trials, and this might weaken the visual similarity effect. Whether the overall pattern of results might have arisen from the use of randomized list types is examined with the blocked list presentation in Experiments 2 (in Japanese) and 3 (in English).

## Experiment 2

The purpose of this experiment was to investigate whether the zig-zag serial position curves for alternating lists with the visual similarity manipulation would appear using a blocked design (Henson et al., 1996). This design should reduce variability in the data that might arise from variations in strategy use by participants across trials. We also increased the number of trials for each list and decreased the number of different types of lists. Together, these changes should increase the statistical power to detect an effect of visual similarity even when scoring for correct recall, a result that was marginal in Experiment 1. As in Experiment 1, participants were fluent Japanese speakers and materials were Japanese Kanji. The general procedure followed that for Experiment 1 with the exceptions that the combined lists were not included and that participants performed the pure list trials and the alternating list trials in separate blocks following the list administration procedure used by Henson et al. (1996).

### Method

#### Participants

Twenty-four undergraduate and graduate students from Hiroshima University, all native speakers of Japanese, participated in this experiment. The age of participants ranged from 18 to 24 years, with a mean of 20.2 years. None had participated in Experiment 1.

### Design, materials, and procedure

Participants were tested in four experimental blocks: Two blocks of pure lists (one dissimilar block and one similar block) and two blocks of alternating lists, each block consisting of four practice lists and ten experimental lists. Each of the two alternating blocks consisted of five DS alternating lists and five SD alternating lists, and each block employed a fixed set of six Kanji materials, three from dissimilar and three from similar sets. The Kanji materials were those used in Experiment 1, with the exception of one replacement in the visually dissimilar set for better control of Kanji frequency (see note for Table 1). The order of the four blocks was counterbalanced across participants; half of the participants performed a pure list block first, with half of those performing the dissimilar block first, and the remainder performing the similar block first. The pure list blocks and the alternating list blocks appeared in turn. The general procedure for the memory task was similar to that in Experiment 1, except that before performing each test block, participants were presented with a list of the six Kanji characters that were to be used in that block in order to reduce item errors, and make the task maximally sensitive to order memory. Concurrent articulation was again used in this experiment.

### Results

We employed the same three scoring methods and similar statistical analyses to those used in Experiment 1.

#### Correct recall

Table 2 shows the mean proportion of correct recall as a function of serial position in each list of four list types. We conducted a two-way ANOVA, with visual similarity factor (two levels: dissimilar vs. similar) and list structure factor (two levels: pure and alternating lists). Mean scores were .63, .57, .60, and .55, respectively, for Dissimilar-Pure, Similar-Pure, Dissimilar-Alternating, and Similar-Alternating lists. The main effect of similarity was significant, indicating better recall performance for dissimilar compared to similar items. Neither the main effect of list structure nor the interaction between the two factors was significant.

#### Item errors

Table 4 shows the mean proportion of item errors as a function of serial position in each list of four list types. A two-way ANOVA, with visual similarity (two levels: dissimilar vs. similar) and list structure (two levels: pure and alternating lists) showed a significant main effect of list structure. Neither the main effect of visual similarity nor the interaction between the two factors was significant. The mean proportions of item errors for Dissimilar-Pure, Similar-Pure, Dissimilar-Alternating, and Similar-Alternating lists were .05, .05, .07, and .07, respectively.

#### Order errors

**Fig. 2:**
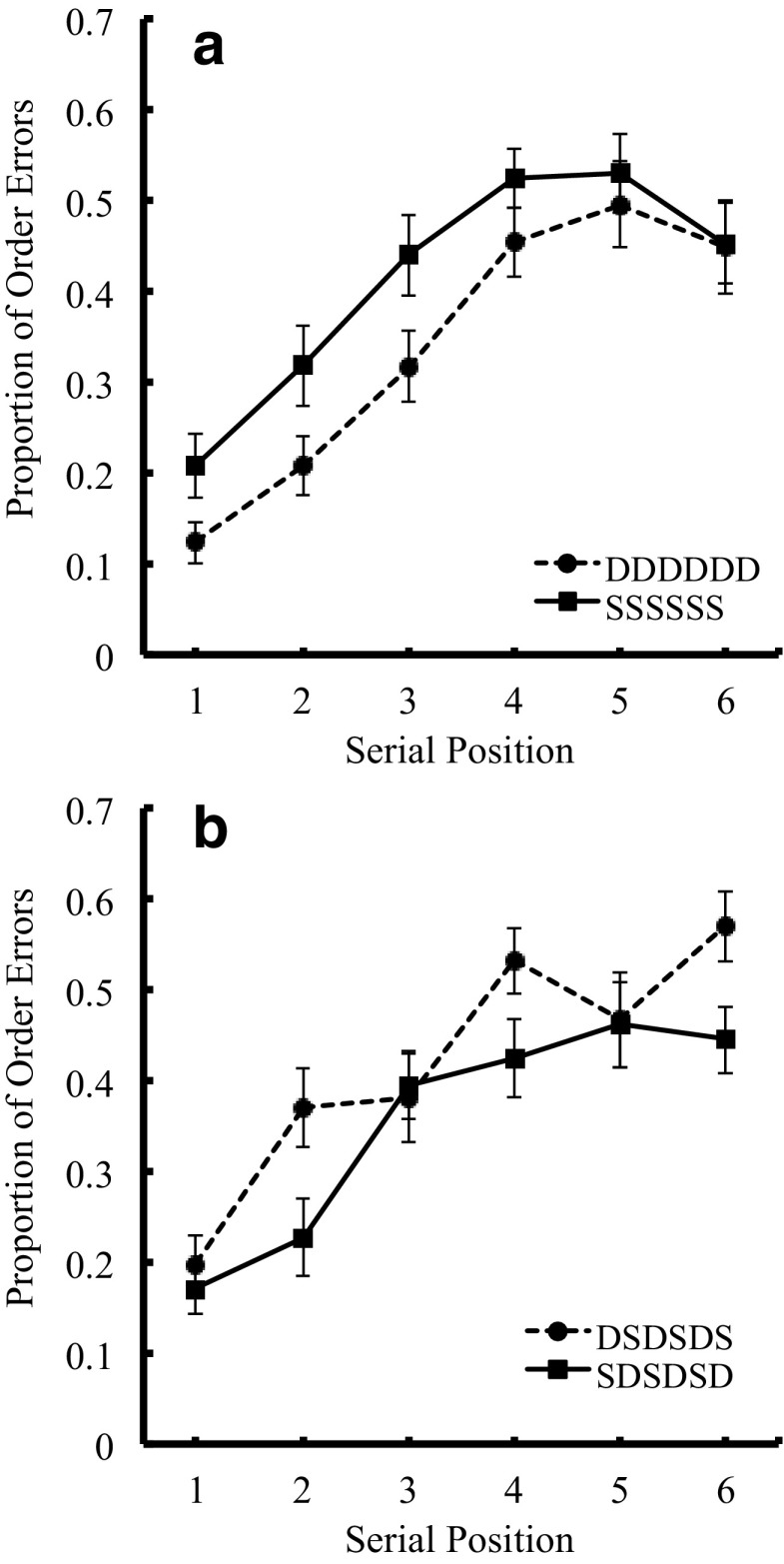
Serial position curves in the proportion of order errors in Experiment 2, as a function of visual similarity for pure lists (Fig. 2a) and for alternating lists (Fig. 2b)

Figure 2a shows serial position curves for the proportion of order errors of the pure lists, and Fig. 2b shows those of the alternating lists (DS alternating and SD alternating lists). A two-way ANOVA, with visual similarity (two levels: dissimilar vs. similar) and list structure (two levels: pure and alternating lists) revealed a significant main effect of similarity, indicating better recall performance for dissimilar over similar items. Neither main effect of list structure nor the interaction between the two factors was significant. The mean scores for Dissimilar-Pure, Similar-Pure, Dissimilar-Alternating, and Similar-Alternating lists were .34, .41, .36, and .42, respectively.

We conducted a two-way ANOVA, with list type (DS alternating vs. SD alternating lists) and serial position (1–6), that showed a significant interaction between the two factors. Table 5 shows the results of the ANOVA that confirmed the presence of saw-tooth serial position curves.

### Discussion

This experiment again demonstrated zig-zag serial position curves for the alternating lists, confirmed by poorer order recall performance of visually similar items than dissimilar items. The lack of an interaction between similarity and list type suggests that the similarity effect was as robust for the pure lists as it was for the alternating lists. Moreover, the similarity effect appeared only for the correct recall and order error scores, but not for the item error score. This indicates that the effect is not solely based on memory for items, and is consistent with the results reported in our previous studies (Logie et al., 2000 in English; Saito et al., 2008 in Japanese). This data pattern matches that observed with manipulations of phonological similarity (similar vs. dissimilar) and list structure (DS vs. SD alternating lists) in Baddeley (1968) and Henson et al. (1996). Although the size of the visual similarity effect (η^2^_G_ = .033 for correct recall; η^2^_G_ = .044 for order errors) here is again smaller than for the phonological similarity effect in the previous studies, the blocked list presentation method (and possibly the simple experimental design, thus increasing the number of trials in each condition) seemed to stabilize the visual similarity effect for the scores involving order, but not item errors, and led to the significant main effect of visual similarity for the pure lists and the alternating lists in both correct recall and proportion of order errors. Given the use of blocked lists, the results are unlikely to be due to differential strategy use across trials within a block.

## Experiment 3

The purpose of Experiment 3 was primarily to assess whether the results obtained in Experiments 1 and 2 are specific to Japanese Kanji, or if they would also be obtained using the alphabetic script of printed English. The general procedure followed that for Experiment 2 in which the visual similarity effect in the pure lists and the alternating lists were reliably observed.

### Method

#### Participants

Twenty-four undergraduate and graduate students (11 male, 13 female) from the University of Edinburgh, all native speakers of English, participated in this experiment. Their age ranged from 22 to 30 years, with a mean of 24.8 years. None of them had participated in the previous experiments.

#### Design, materials, and procedure

The experimental design was identical to that for Experiment 2, but the stimuli were phonologically similar English words drawn from Logie et al. (2000) to give four list structures: SSSSSS, DDDDDD, SDSDSD, and DSDSDS. There were two pools of six words. One pool had only visually similar words: CRY, DRY, TRY, SHY, PLY, FLY. The other pool took advantage of irregular spelling to sound correspondence in English to be visually distinct: THAI, HI, LIE, SIGH, GUY, RYE. The choice of phonologically similar words across lists helped control for possible differences in phonological similarity between lists, and undermined the utility of phonological codes in memory for each presented list, so as to encourage the use of visual codes. The mean frequency of the dissimilar words was lower than that of similar words. However, low word frequency is typically associated with poorer recall, and here we are expecting poorer performance for the visually similar list, so the mismatch in frequency was conservative with respect to our hypotheses. Nevertheless, the words were familiar to participants. The only change from the original Logie et al. (2000) study was to replace the word PI with HI, as pilot studies showed that subjects often confused it with seeing the word PIE which was strikingly similar to the word LIE and therefore not appropriate for the visually distinct set.

Participants were tested in four experimental blocks: Two blocks for pure lists (one dissimilar block and one similar block) and two blocks for alternating lists; each block consisted of ten test lists and four practice lists. Each of the two alternating blocks consisted of five DS alternating lists and five SD alternating lists, and each block employed a fixed set of six words, three from dissimilar and three from similar sets. The testing order of the four blocks was counterbalanced across participants as in the second experiment.

The concurrent articulation procedure involved participants uttering aloud “the, the, the” from the onset of a fixation point, which indicated the start of the trial, until presentation of a question mark, which indicated the start of the recall period. The procedure for concurrent articulation was identical to Experiments 1 and 2, except that the language used was English rather than Japanese and that it was administered only during item presentation in contrast to the first two experiments in which participants engaged in suppression during both item presentation and recall. The purpose of the inclusion of the suppression was to minimize the use of phonological codes. It has been established that the influences of phonological codes are completely removed when concurrent articulation is required during visual item presentation in English speaking participants (e.g., Baddeley, Lewis & Vallar, 1984). Therefore this methodological change should not affect the examination of visual similarity effects.

At the beginning of each list, a message (white on black background) on the screen reminded the participants to begin articulating “the, the, the” at the rate of 3 per second and press “Enter” when they were ready. All stimuli were presented in an MS Serif font size 18 for instructions and 24 for stimulus words. The presentation began with a fixation cross appearing at the center of the screen for 3 s. As in Experiment 1, list items were presented at a rate of one every second. At the offset of the last word, the screen turned black, at which point the participant was required to write the words in the exact order they were presented in boxes marked on the sheet provided. There was no limit on the time taken for recall; however, participants were encouraged to complete the process within 60 s. The response sheet was removed at the end of recall and participants could proceed to the next trial by pressing the “SHIFT” key.

Participants were instructed to remember the words visually by concentrating on the visual appearance of the letters. They were asked not to use any phonological, associative, semantic, or linguistic prompts with the words or memorize on the first letter basis. Further they were required to write their recall in strict left-to-right fashion.

### Results

We employed the same three scoring methods and similar statistical analyses as for the previous two experiments.

#### Correct recall

Table 2 shows the mean proportion of correct recall as a function of serial position in each list of the four list types. We conducted a two-way ANOVA with visual similarity (two levels: dissimilar vs. similar) and list structure (two levels: pure and alternating lists). Mean scores were .53, .47, .56, and .42, respectively, for Dissimilar-Pure, Similar-Pure, Dissimilar-Alternating, and Similar-Alternating lists. The main effect of similarity was significant, indicating better recall performance for dissimilar over similar items. The main effect of list structure was not significant but the interaction between the two factors was significant with the difference between dissimilar items and similar items being smaller in the pure list (.06) than that in the alternating lists (.14). The visual similarity effects were significant in both type of lists (*p* = .004 for the pure lists and *p* < .001 for the alternating lists).

#### Item errors

Table 4 shows the mean proportion of item errors as a function of serial position in each list of four list types. A two-way ANOVA, with visual similarity (two levels: dissimilar vs. similar) and list structure (two levels: pure and alternating lists) showed a significant main effect of visual similarity, indicating better item recall for the dissimilar over the similar items. The main effect of list structure was also significant as was the interaction between the two factors. Although the difference in the proportion of errors between dissimilar items and similar items in the alternating lists (.13) was significant (p < .001, d = .923), that in the pure lists (−.02) was not significant (n.s., d = .292). The mean proportions of item errors for Dissimilar-Pure, Similar-Pure, Dissimilar-Alternating, and Similar-Alternating lists were .23, .21, .25, and .38, respectively.

#### Order errors

**Fig. 3:**
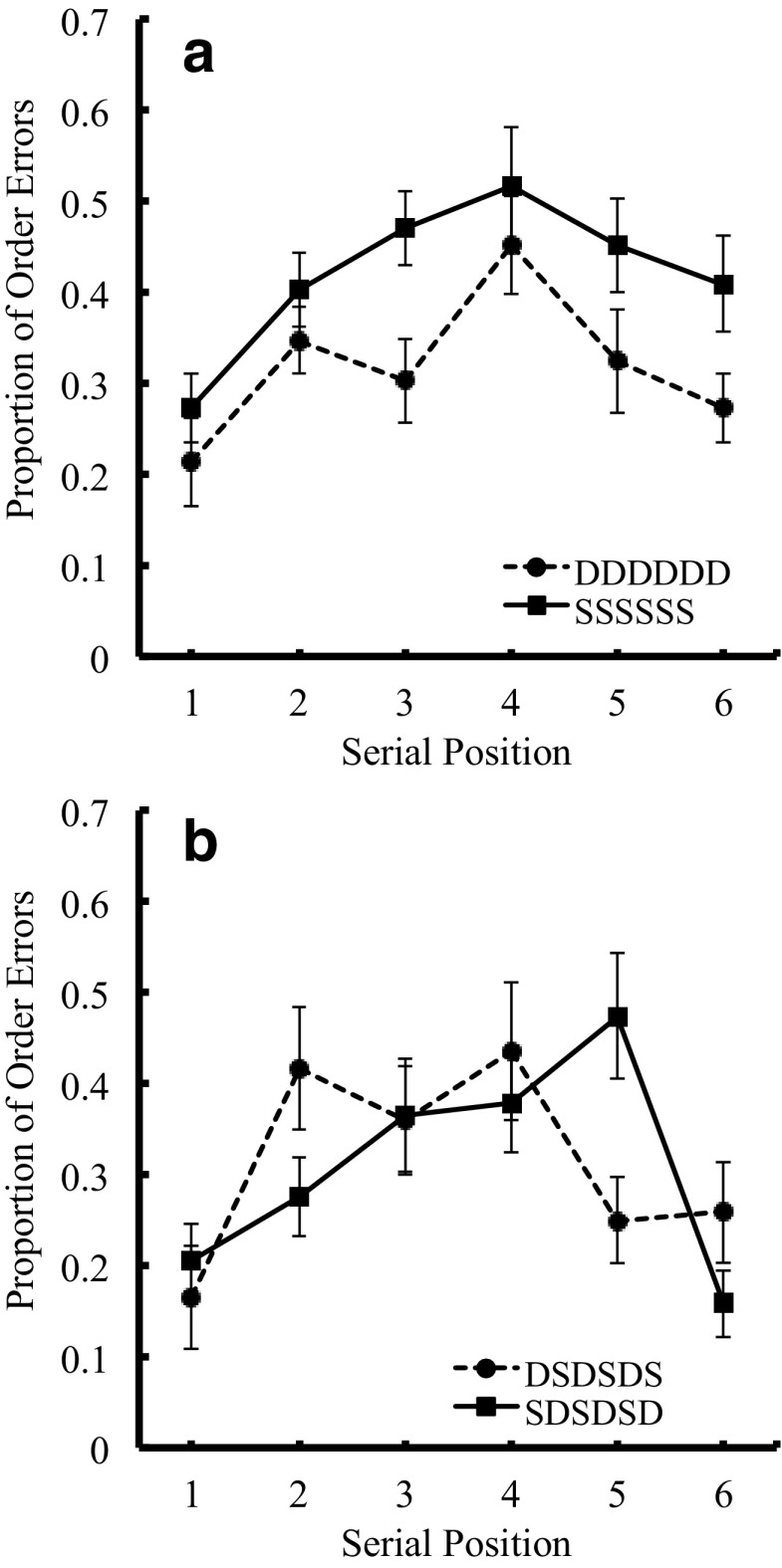
Serial position curves in the proportion of order errors in Experiment 3, as a function of visual similarity for pure lists (Fig. 3a) and for alternating lists (Fig. 3b)

Figure 3a shows serial position curves for the proportion of order errors in the pure lists, and Fig. 3b shows those of the alternating lists (DS alternating and SD alternating lists). A two-way ANOVA, with visual similarity factor (two levels: dissimilar vs. similar) and list structure factor (two levels: pure and alternating lists) revealed a significant main effect of similarity, indicating better recall performance for dissimilar over similar items. The main effect of list structure was marginally significant. The interaction between the two factors was not significant. Mean proportions of order errors for Dissimilar-Pure, Similar-Pure, Dissimilar-Alternating, and Similar-Alternating lists were .32, .42, .26, and .36, respectively.

We conducted a two-way ANOVA, with list type (DS alternating vs. SD alternating lists) and serial position (1–6), resulting a significant interaction between the two factors. Table 5 shows the results of the ANOVA and confirmed the saw-tooth serial position curves.

### Discussion

We obtained a significant effect of visual similarity overall in the pure lists, again replicating the results of the Logie et al. (2000) and Saito et al. (2008) studies, which showed a visual similarity effect under concurrent articulation. Furthermore, in a clear parallel with the results of the Baddeley (1968) and Henson et al. (1996) studies of phonological similarity, we obtained new data showing the zig-zag serial position curves for the lists alternating visually similar and visually distinct items. Crucially, the effects of visual similarity were present even when scoring for order errors, suggesting that the effect was not solely based on memory for items. Together with the data from Experiments 1 and 2 with Japanese participants, we have shown that the visual similarity effect in serial order recall behaves like the effect of phonological similarity in alternating lists, suggesting the presence of similar processes for the retention of phonological serial order and that of visual serial order.

## Simulation: An illustration with the Primacy Model

We further investigated the similarity of the behavioral data patterns of visual serial recall of verbal material by exploring whether the data from one of our experiments (Experiment 3) could be simulated by a computational model of serial-ordered recall. As noted earlier, there are several different computational models available. We chose the Primacy Model (Page & Norris, 1998), in part because it makes no assumptions about whether phonological or visual codes are used by participants, and because it was one of the first models to simulate the zig-zag effect for phonological similarity reported by Henson et al (1996). It also represents serial order as different levels of activation of each item in the list, so there is no requirement to assume a serial order mechanism that is separate from the memory system that stores the items. Our aim was to illustrate that our data can be simulated by at least one model thereby indicating that our new data on the use of visual codes for immediate serial recall of verbal items are comparable with data reported in multiple previous studies that have focused on the use of phonological codes in equivalent tasks.

The Primacy Model’s ability to simulate serial recall data stems from a small number of simple assumptions. The first is that the relative order of items can be represented by their activation levels. Activation levels form a primacy gradient where the first item in a sequence has the highest activation and subsequent items have progressively lower activations. Activations decline, either because of the passage of time or because of interference from subsequent items. Recall takes place by adding noise to the activation levels of each item, selecting the item with the largest activation, and then suppressing that item so that it is not recalled again. Because the activation decays over time the difference in activation levels between items decreases as list recall proceeds, so performance also decreases. This produces a primacy effect. However, as more items are recalled, there are fewer competing items remaining from which to select. This produces an increase in performance towards the end of the list leading to a recency effect. In serial recall, the recency effect is always much smaller than the primacy effect. A simple way to characterize the operation of the model is to say that the signal-to-noise ratio of order information declines with time. Although other models use different algorithms, the high-level principles are very similar. The way the model accounts for phonological similarity effects is slightly more complicated. For the present purpose the critical feature of the model is that similar items activate each other in such a way that they are more likely to swap positions with each other than are dissimilar items. Given that the model does not use any form of chaining, and that inter-item similarity increases order errors, the model readily accounts for the zig-zag pattern seen with alternating lists of phonologically similar and phonologically different items.

Note that several other computational models of short-term memory can also simulate the verbal short-term memory data, and we have no doubt that they could be fitted to our new data. Here we simply demonstrate that a model that was developed to simulate verbal short-term memory can fit data from visual short-term memory without modification. The Primacy Model has six free parameters: Peak activation level, decay rate, selection noise in the first level, selection noise in the output level, similarity between similar items in the second level, omission threshold, and noise in the omission threshold (see Page and Norris, 1998, for a detailed explanation of these parameters). In accordance with the presentation timing in the experiment, the inter-onset interval in the model was set to 1 s.

Because the trials for pure lists in Experiment 3 were completely blocked and separated from the alternating lists, the results of the pure and alternating conditions were fitted separately. Moreover, the contrast between pure lists is demonstrated between blocks whereas that of alternating lists is necessarily within each list.

These parameters were optimized to minimize the root-mean-square (RMS) error between the simulated recall accuracy and the data using Powell’s conjugate gradient method (Powell, 1964; Press, Teukolsky, Vetterling, & Flannery, 1995). At each optimization step the model was run 100,000 times, which is more than sufficient to achieve stable and replicable results.

### Demonstration 1

**Fig. 4:**
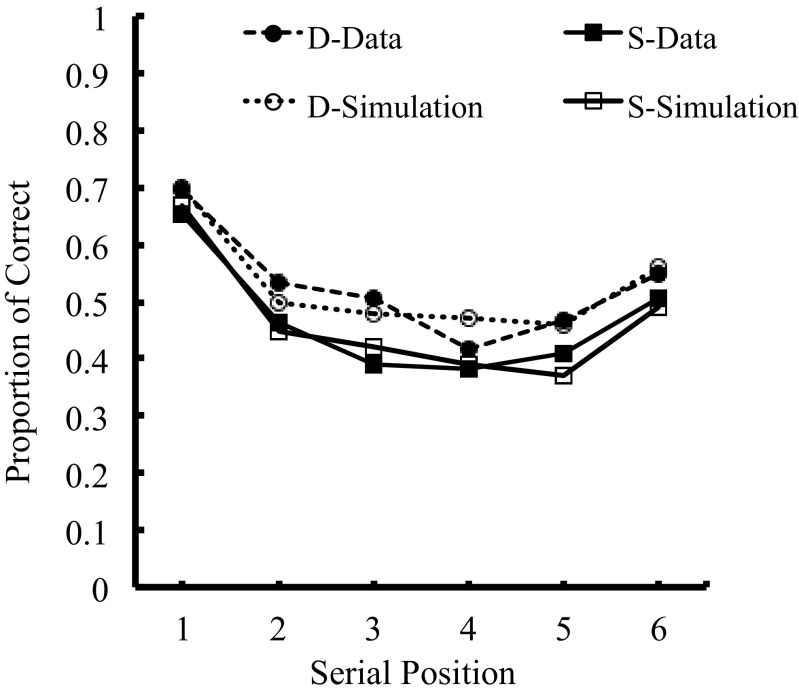
Primacy Model simulation showing plots of fits on pure-list data in Experiment 3

Figure 4 shows the simulation for the pure lists: SSSSSS and DDDDDD, with overall RMS error of 0.025. Note that all comparisons between the human data and the model use correct recall scoring. This figure clearly demonstrates that the model produced good fits to the data from Experiment 3 showing a visual similarity effect across all serial positions.

### Demonstration 2

**Fig. 5:**
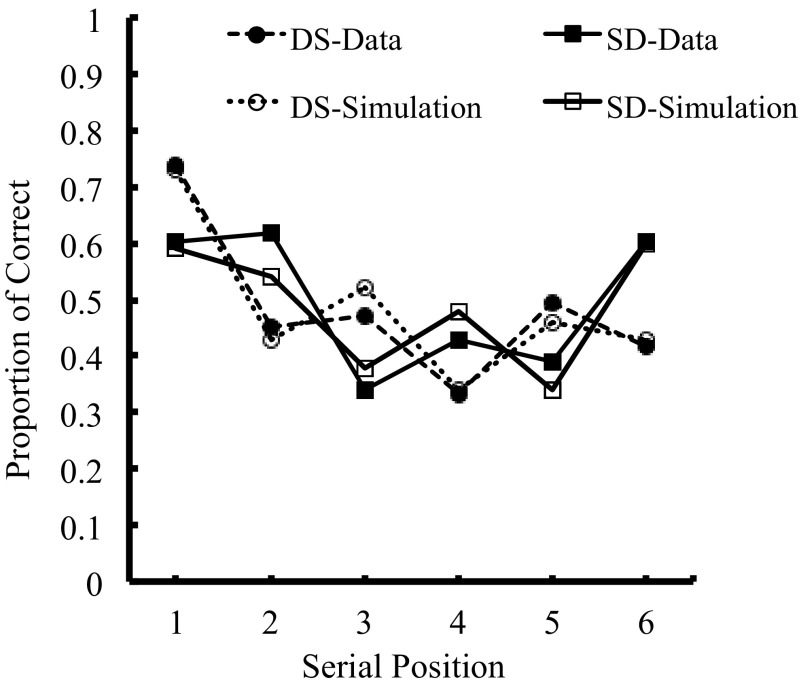
Primacy Model simulation showing plots of fits on alternating-list data in Experiment 3

Similarly, the alternating lists simulation, shown in Fig. 5, is consistent with our data (correct recall scores) from Experiment 3 and produces an RMS error of 0.036. Once again, the Primacy Model demonstrates that in alternating lists, more errors occur for the visually confusable items than for the visually dissimilar items.

The Primacy Model is clearly capable of producing good fits to the data. However, most importantly, this is achieved without any additional mechanism or parameters. Although phonological and visual similarity effects do not originate from the same forms of memory code, the data fits with the Primacy Model provide evidence that they show broadly the same serial recall characteristics, without the need to assume (e.g. Brown et al. 2000; Jones, Macken & Nicholls, 2004; Depoorter &Vandierendonck, 2009) an amodal system for serial recall that is separate from temporary memory systems for items.

## General discussion

We examined the nature of visual memory for serially ordered verbal information in three experiments on immediate written serial recall of Japanese Kanji characters (Experiments 1 and 2) and English monosyllabic words (Experiment 3), contrasting recall of visually similar and visually dissimilar items. In the first experiment, in which list type conditions were presented in random order within a single block, the visual similarity effect in order errors was found to be reliable and did not significantly differ across the different list types, namely pure lists (SSSSSS or DDDDDD) alternating lists (SDSDSD and DSDSDS), and combined lists (SSSDDD and DDDSSS). When the pure lists and the alternating lists were performed in separate blocks in Experiments 2 and 3, we confirmed the significant effect of visual similarity across the pure lists. Crucially, we found zig-zag serial position curves in the alternating lists in all three experiments, and the poorer recall for the visually similar items than for the visually dissimilar items across experiments was more reliable for serial order than for item memory. We also presented simulations of the data from Experiment 3 using the Primacy model (Page and Norris, 1998) to illustrate that a model developed specifically to account for phonological memory could readily fit our data on visual-verbal memory. These findings substantially extend previous results (Logie et al., 2000; Saito et al., 2008) demonstrating for the first time the presence of similar mechanisms for immediate visual serial-ordered recall as has been found previously only for immediate phonological serial-ordered recall. The new experiments also demonstrate this in two very different languages, suggesting that our results are not simply due to selection of idiosyncratic materials with items that are easier to recall in serial order independently of their visual dissimilarity. The less stable effects found in Experiment 1 seem to indicate that participants may flexibly select a visual code, a phonological code, or another code depending on the experimental block structures, reinforcing the possible role of strategy in serial-ordered recall tasks (Logie et al., 1996; Johnson, Logie & Brockmole, 2010). This is consistent with the Saito et al. (2008) demonstration of effects of concurrent articulation on memory for phonologically based serial order but not for visually based serial order, suggesting that they involve distinct memory mechanisms. Our new results suggest that these two different memory systems operate according to similar principles. In the following discussion, two issues will be addressed. We will discuss first the role of visual codes in retention of serial order of verbal materials and then the mechanisms of serial order memory.

### The role of a visual code and its relation to a phonological code in verbal immediate serial recall

The fact that the effect of visual similarity was observed across list types and across all conditions indicated use of visual codes within a list, as suggested by previous studies (Logie et al., 2000; Saito et al., 2008). One of the important findings from the current study is that the pattern of results was similar for both the Japanese and British participants despite the difference in memory materials (i.e., Japanese Kanji characters vs. alphabetical letters). The list structure (pure or alternating lists) showed similar patterns of visual similarity in both Japanese and British groups, indicating the universality of the effect and its underlying mechanisms.

A fundamental question here is what kind of mechanisms might lead to a visual similarity effect in verbal immediate serial recall, and, more generally, how the visual code for the verbal materials supports performance of immediate serial-ordered recall.

One possibility might be that visual features could act only as supplementary information to discriminate among the verbal items presented. In one version of this account, it might be possible to assume that only a phonological code can retain serial order information, but that visual as well as phonological codes could be used in the process of redintegration at recall. However, although visual redintegration might have occurred, we do have to consider that a visual code itself could maintain serial order information. Furthermore, we found a visual similarity effect under concurrent articulation in the current three experiments, as well as in Saito et al. (2008) and Logie et al. (2000) suggesting that the retention of serial order does not depend on the use of a phonological code. Crucially, the visual similarity effect remained even when scoring only for order of recall. We also reduced the possible contribution from item memory by using the same items repeatedly in different orders across trials. Therefore, it seems reasonable to suggest that visual codes were being used to retain serial order. Smyth et al. (2005) reported that serial order memory for faces showed serial position curves similar to that found for verbal materials, and it seems unlikely that a phonological code played a major role in retaining faces. However, a key difference with the use of faces as stimuli is that retrieval is tested typically either by probed recognition, or by serial order reconstruction from an array presented at test. The latter is also true when testing serial order memory for visual patterns or for Corsi block sequences (e.g., Logie & Pearson, 1997; Milner, 1971; Vandierendonck, Kemps, Fastame, & Szmalec, 2004) in which the items are presented at study and at recall but it is order that has to be recalled. In the case of the verbal material used here, memory is tested by written recall and so participants have to regenerate all of the items in the original order of presentation, and in a visual-motor form rather than by spoken recall. The latter form of output is common in verbal serial recall tasks and spoken output might encourage participants to rely more heavily on phonological codes, while reducing the utility of visual codes for meeting task requirements. The contrast between these different modes for testing immediate memory for serial order for visual and for phonological codes would be an interesting topic for future research in this area, and might help address the influence of output interference relative to sources of forgetting in the memory system.

As Avons (1998; Avons & Mason, 1999) and Smyth et al. (2005) indicated, the same mechanism might support retention of serial order regardless of whether the material is visual or phonological. Farrand and Jones (1996; see also Vandierendonck et al., 2004) reported that recall patterns for visual material and verbal material are similar for forwards and backwards serial recall. They argued that neither a visual nor a phonological code directly supports the retention of serial order, but a common mechanism could retain serial order of both visual and phonological memory materials. This assumption would seem to predict that any experimental variables that negatively affect the retention of serial order for one type of material would necessarily negatively affect memory performance on serial order recall of other types of material. Therefore we might expect that manipulations affecting serial order based on phonological codes, as indicated by the presence of a phonological similarity effect, should also affect serial order based on visual codes, as indicated by the presence of a visual similarity effect. Consistent with this expectation, Jones, Farrand, Stuart and Morris (1995) reported that immediate serial-ordered recall of the locations of a dot sequence was disrupted by mouthed articulatory suppression and irrelevant speech, similar to the disruption observed by serial-ordered verbal recall. This result was replicated recently by Guitard and Saint-Aubin (2015). However, this expectation has difficulty accounting for findings that show differential effects of disruption on visual and verbal serial recall. As noted earlier, Saito et al. (2008) showed that concurrent articulation completely eliminated the phonological similarity effect while leaving the visual similarity effect intact. In particular, concurrent articulation did not have an impact on visual similarity when recall was scored for order, but did disrupt memory for phonologically based order. This evidence is not consistent with the suggestion that visual and phonological codes both rely on a common, amodal retention system for serial order information.

As Logie et al. (1996) demonstrated, participants may vary the strategy that they use in verbal serial recall, and so whether or not a particular task disrupts recall performance may depend on how participants are performing the task. For example if participants support the recall of spatial locations with the use of verbal codes to count the number of dots or approximately where the dots are on the screen, then a concurrent verbal task is likely to be disruptive (e.g., Logie & Baddeley, 1987). If participants rely on a visual code for the task, then a concurrent verbal task may be ineffective, as appeared to be the case in the Logie et al. (2000) and Saito et al. (2008) experiments. In studies reporting very similar results for visual and for verbal material, rarely are there reports of the kinds of errors that participants generate. For example, if participants fail to show visually based errors in recall of visually presented material, this might suggest that they are not using visual codes for retention in memory. As we have argued here, showing similar results for serial-ordered recall of visually presented material as has been shown for serial-ordered recall of verbal material, without demonstrating that different types of memory codes were used for each does not unambiguously lead to the conclusion that a common serial order mechanism is in use, particularly given the neuropsychological dissociations as well as the experimental dissociations that have been observed in previous studies. Similar findings for serial-ordered recall across different types of material may simply suggest that participants are using similar memory codes for both types of material. Therefore, the idea of separate, domain-specific temporary memory systems that function in similar ways to support serial order can account for both the appearance of similar data patterns and for differential data patterns in serial-ordered recall studies across different modalities. The idea of an amodal serial order system can account for the former but not the latter set of findings.

Other data that appear to be inconsistent with our conclusion were reported by Depoorter and Vandierendonck (2009) who showed cross-modality interference when both verbal and non-verbal sequence information had to be retained, but a lack of cross-modal interference when only item information had to be retained. They concluded that this suggested a common serial order mechanism. However, if each domain-specific memory system codes serial order for the items it retains, then during output there could be confusion regarding from which memory system items should be selected for serial recall. That is, the Depoorter and Vandierendonck result could arise from output interference when attempting retrieval from different memory systems, each of which is retaining a different serial order for different item sets, rather than because of the use of a common serial order system for both sets of items. A more recent study by Farrell and Oberauer (2014) contrasted serial order for mixed-modality lists (visual, spatial, and verbal stimuli) compared with lists within only one modality (e.g., only visual). They reported an advantage for mixed-modality lists over pure modality lists, and argued that this finding supported the view that there is a domain general memory and serial order mechanism. However, because they used the visual modality for presentation and visuo-motor serial reconstruction to test recall, participants could well have used the same modality-specific memory system for all three types of stimulus material. Therefore, the mixed lists could have been superior because of the use of more distinctive features across lists within a modality-specific system.

More consistent with the evidence we report here and in our previous studies is the view that retention of serial order arises from common processes that are used in separate, domain-specific temporary memory systems. The Primacy Model offers one way of characterizing such systems, with serial order represented as differential levels of activation within each memory system. One possibility is that the zig-zag pattern is not a characteristic peculiar to verbal or phonological memory, but reflects a fundamental property of memory that holds across all domains (e.g., Jackendoff, 2002). For example, in a short-term memory experiment it would be possible to store the list of digits “2,6,3,9,5” by activating the long-term memory representation of each digit as a function of position. But this would not work for a list where two of the items are the same: “2,6,3,3,5.” Moreover, if the same items are repeatedly presented across trials but in different orders, the build-up of proactive interference across multiple trials would undermine the utility of this form of item-based memory. The standard solution to this problem is to suggest that instead of having a single “type” representation for each digit in memory, there should be dynamically assignable representations of individual tokens (e.g., Bowman & Wyble, 2007; Kanwisher, 1991). This might, for example, take the form of pointers in a short-term memory system. Of course, those tokens themselves need to be associated with positions that are different on every trial. What is required then is to bind items to positions. This requirement applies equally to verbal and visual sequences. In terms of the computations required, binding features representing items with their position in a sequence would be no different than binding, say, a shape with a color or with a location in a stimulus array. The task of remembering serial order of items that are adjacent or further apart in a list then becomes analogous to remembering the location of items that were in close proximity or were in more clearly different spatial locations in the visual array. Location is viewed as one of multiple stimulus features that form the integrated representation of an object in visual short-term memory (e.g., Logie, Brockmole & Jaswal, 2011; Wheeler & Triesman, 2002). When participants have to remember color-shape-location combinations, errors are often the result of remembering a shape or color as being presented in a different location, or a color being presented with a different shape (e.g., Gajewski & Brockmole, 2006). In memory for an array, we would expect that items in close spatial proximity would be more likely to swap positions in the memory representation. Viewed in this way, an order error in serial recall is an error in memory for the binding of an item to its position in the presented sequence; items become bound to the wrong positions in the memory representation. In serial recall the items most likely to exchange position are those that have the most phonologically similar representation, and this would be true of the visually similar items for the current experiments and in both of our own previous experiments (Logie et al., 2000; Saito et al, 2008), as well as in other studies (Smyth et al. 2005; Walker et al., 1993). The zig-zag pattern suggests that swapping of serial position primarily involved the visually similar items, leaving the dissimilar items largely unaffected. According to this view, therefore, the zig-zag pattern in previous studies of phonologically based immediate serial-ordered recall (Baddeley, 1968; Henson et al. 1996), and in our new studies of immediate visually based serial recall is a consequence of errors in binding items and positions, and this is modulated by phonological similarity or by visual similarity of the items. The zig-zag pattern emerges simply as a consequence of noise in the binding process within modality-specific memory systems. This interpretation, although somewhat speculative, offers a range of hypotheses for future empirical studies that could help integrate research on visual short-term memory for feature binding with research on serial-ordered recall.

In principle then, any memory system might function in a similar manner when given the task of retaining serial order. In this case, we could assume that patterns of memory performance for visual and phonological materials might be very similar, but that there could be situations in which experimental manipulations might differentially affect serial order memory performance for different sets of materials. As Smyth et al. (2005) and Saito et al. (2008) suggested, any system supporting memory for serial order might show characteristic serial position curves and effects of within-list item similarity, even if there are separate, modality-specific temporary memory systems. Our new data also support this idea.

## Summary and conclusion

Across three experiments, we have demonstrated the use of visual codes for immediate serial-ordered recall of visually presented verbal material, under concurrent articulation. The patterns of recall were similar to those found in previous studies for immediate verbal serial recall based on phonological codes. The results also show a close fit with at least one computational model for short-term serial recall, the Primacy Model. Taken together with previous findings on this topic, the data patterns are most readily explained by assuming separate temporary memory systems for visual and for phonological material that employ similar processes to retain serial order.
